# Molecular Classification of Endometrial Cancer and Its Impact on Therapy Selection

**DOI:** 10.3390/ijms25115893

**Published:** 2024-05-28

**Authors:** Natalia Galant, Paweł Krawczyk, Marta Monist, Adrian Obara, Łukasz Gajek, Anna Grenda, Marcin Nicoś, Ewa Kalinka, Janusz Milanowski

**Affiliations:** 1 Department of Pneumonology, Oncology and Allergology, Medical University of Lublin, 20-090 Lublin, Poland; nataliagalant@umlub.pl (N.G.); pawel.krawczyk@umlub.pl (P.K.); marcin.nicos@umlub.pl (M.N.); janusz.milanowski@umlub.pl (J.M.); 2 II Department of Gynecology, Medical University of Lublin, 20-090 Lublin, Poland; marta.monist@umlub.pl; 3 Institute of Genetics and Immunology GENIM LCC, 20-609 Lublin, Poland; obara12lb@interia.pl (A.O.); luk.gajek@gmail.com (Ł.G.); 4 Department of Oncology, Polish Mother’s Memorial Hospital-Research Institute, 93-338 Łódź, Poland; ewakalinka@wp.pl

**Keywords:** endometrial cancer, molecular subtypes, personalized treatment

## Abstract

Endometrial cancer (EC) accounts for 90% of uterine cancer cases. It is considered not only one of the most common gynecological malignancies but also one of the most frequent cancers among women overall. Nowadays, the differentiation of EC subtypes is based on immunohistochemistry and molecular techniques. It is considered that patients’ prognosis and the implementation of the appropriate treatment depend on the cancer subtype. Patients with pathogenic variants in *POLE* have the most favorable outcome, while those with abnormal p53 protein have the poorest. Therefore, in patients with *POLE* mutation, the de-escalation of postoperative treatment may be considered, and patients with abnormal p53 protein should be subjected to intensive adjuvant therapy. Patients with a DNA mismatch repair (dMMR) deficiency are classified in the intermediate prognosis group as EC patients without a specific molecular profile. Immunotherapy has been recognized as an effective treatment method in patients with advanced or recurrent EC with a mismatch deficiency. Thus, different adjuvant therapy approaches, including targeted therapy and immunotherapy, are being proposed depending on the EC subtype, and international guidelines, such as those published by ESMO and ESGO/ESTRO/ESP, include recommendations for performing the molecular classification of all EC cases. The decision about adjuvant therapy selection has to be based not only on clinical data and histological type and stage of cancer, but, following international recommendations, has to include EC molecular subtyping. This review describes how molecular classification could support more optimal therapeutic management in endometrial cancer patients.

## 1. Introduction

Endometrial cancer (EC) is caused by the growth of the mucous membrane lining the uterus [1], and, along with rarer uterine sarcoma, it is classified as a type of uterine cancer. Endometrial cancer accounts for 90% of uterine cancer cases [2,3] and is considered not only one of the most common gynecological malignancies [4,5,6] but also one of the most frequent cancers among women overall [1,4,7]. Moreover, EC incidence and mortality are still increasing, and further increases are predicted for the coming decades [8,9]. Thankfully, recent progress in the knowledge about EC gives hope for determining the optimal therapeutic management in patients, thereby avoiding under- or over-treatment, which may translate into better quality and comfort in their lives [9,10].

In the past, endometrial cancer was usually classified as one of two types, depending on different histology and patient outcomes. Type I was characterized as mainly estrogen-dependent, occurring more often but with a lower tumor grade and better prognosis. Type II was identified as cancer not associated with estrogen exposure, occurring less frequently, although with a worse prognosis and higher mortality [11,12,13]. The genetic background of EC, including its association with Lynch syndrome, was also considered during diagnostics [14].

However, due to TCGA (the Cancer Genome Atlas), the molecular characterization of EC published in 2013, different classifications of this cancer type are proposed [15]. Endometrial cancer can be identified as one of four molecular subtypes:

- EC with mutations in the gene encoding polymerase epsilon (*POLE*mut);
- EC with deficiency of mismatch repair (dMMR) and high microsatellite instability (MSI-High);
- EC with mutations in the *TP53* gene and abnormal expression of p53 protein (p53abn);
- EC with no specific molecular profile (NSMP).

**Figure 1:**
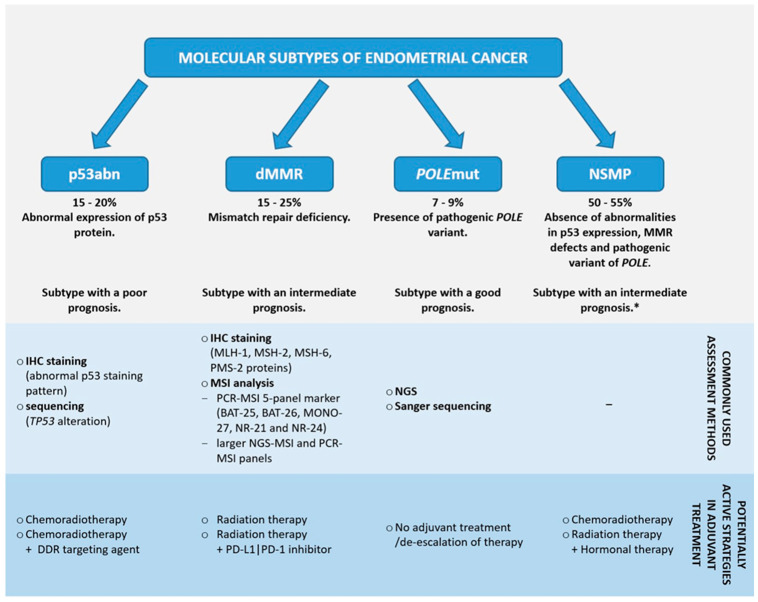
Summary of information regarding endometrial cancer molecular subtypes. The diagnosis of the molecular subtype is hierarchical according to the PROMISE diagram, with *POLE* mutation as the dominant determinant even if p53abn status is found. Based on [16,17,21,22,23,24,25]. * NSMP is sometimes considered a subtype associated with poor prognosis [26,27]. DDR—DNA damage response; IHC—immunohistochemistry; MMR—mismatch repair; dMMR—deficiency of mismatch repair; MSI—microsatellite instability; NGS—next-generation sequencing; *POLE*—DNA Polymerase Epsilon, Catalytic Subunit; NSMP—no specific molecular profile; PD-1—programmed death receptor 1; PD-L1—programmed death-ligand 1.

Nowadays, the differentiation of EC subtypes is based on immunohistochemistry (IHC) and molecular techniques (Figure 1). It is considered that patients’ prognosis and the implementation of appropriate treatment depends on the cancer subtype [16,17]. Patients with the *POLE*mut subtype have the most favorable outcome, while those with the p53abn subtype have the poorest [16]. Different adjuvant therapy approaches, including targeted therapy and immunotherapy, are being proposed depending on the EC subtype. Therefore, the molecular classification of all EC cases is highly recommended [16,17,18]. Scientific research is ongoing to make diagnostics cost-effective and implementable in routine clinic practices [9,17,19,20]. The main aim of this article is to summarize current knowledge about the possibilities of management in each molecular subtype of EC. The article is prepared to indicate possible options during EC diagnostics and therapy decision-making. Moreover, it highlights future perspectives on EC treatment based on ongoing clinical trials.

## 2. Molecular Classification of Endometrial Cancer

Currently, the most common approach to assessing the subtype of endometrial cancer is to perform both immunohistochemistry and molecular examinations of tumor tissue [15,17]. IHC enables the detection of abnormal p53 expression and mismatch repair deficiency, while PCR (polymerase chain reaction) and sequencing methods are used to identify pathogenic *POLE* mutations and the presence of MSI [16,17]. PCR involving the separation of reaction products in capillary electrophoresis or next-generation sequencing (NGS) is potentially valuable for the analysis of microsatellite instability (MSI), which is the effect of MMR deficiency [27,28]. Moreover, NGS techniques enable the assessment of *TP53* mutation status, which may be useful during p53abn subtype differentiation, rather than IHC staining [20,29]. However, the panel of the College of American Pathologists experts recommended using IHC for dMMR detection over MSI analysis in EC patients [27].

### 2.1. No Specific Molecular Profile (NSMP)

NSMP is a subtype encompassing the largest, most heterogeneous group of EC cases [26,30,31]. NSMP endometrial cancers are characterized by a lack of mismatch repair defects, p53 abnormalities, and pathogenic variants of the *POLE* gene [17,19,32]. Moreover, these tumors are often characterized by high estrogen and progesterone receptor expression [17]. NSMP is mainly considered a subtype with an intermediate prognosis [16,17,33], but is sometimes associated with a poor prognosis [25,26]. NSMP is a problematic subtype as there is no specific predictive biomarker [17]. Markers such as L1-cell adhesion molecule (L1CAM) overexpression, a lack of expression of estrogen or progesterone receptors, β-catenin (CTNNB1) mutations, and the amplification of chromosome 1q are being proposed as potentially applicable to the improvement of NSMP stratification [17,25,26,30]. Thus, the NSMP subtype is probably a heterogenous EC group expected to be subdivided into smaller categories when new evidence is available. The diagnosis of NSMP can be made by excluding the three remaining subtypes and not by confirming a specific biomarker. EC grade, stage, histotype, and lymphovascular invasion are used in therapy decision-making [17,31,32].

### 2.2. dMMR

The MMR system consists of seven proteins essential for identifying and correcting mismatched bases during DNA replication. These proteins are MLH-1 (MutL homolog 1), MLH-3 (MutL homolog 3), MSH-2 (MutS homolog 2), MSH-3 (MutS homolog 3), MSH-6 (MutS homolog), PMS-1 (postmeiotic segregation increased 1), and PMS-2 (postmeiotic segregation increased 2) [28,34]. MLH-1, MSH-2, MSH-6, and PMS-2 seem clinically significant [28,35]. They form two dimers—MSH-2/MSH-6 complex, which recognizes mismatched bases during replication, and MLH-1/PMS-2, which repairs them [34,36]. MMR-deficient (dMMR) due to one or more protein dysfunctions or its low expression leads to DNA repair disorders, causing the accumulation of mutations and microsatellite instability [28,34,35,37].

Microsatellites are short DNA sequences with tandem repeats occurring in the human genome, mostly in non-coding regions. MSI is characterized as changes in microsatellites’ length. High MSI is a marker of predisposition to carcinogenesis, especially of colorectal cancer (CRC), including hereditary non-polyposis colorectal cancer (HNPCC), but also different tumor types, including endometrial cancer [38,39]. The MSI-H (MSI-High)/dMMR phenotype may be present, among others, in patients with Lynch syndrome, which have hereditary cancer predispositions due to the presence of pathogenic variants in genes encoding MMR proteins [27,40,41]. Lynch syndrome, precisely type II, predisposing to cancer formation in extraintestinal tissue [40], seems to be associated with approximately 3% of all EC cases [37]. The dMMR subtype of EC is considered a subtype with an intermediate prognosis [16,33].

#### dMMR and MSI Detection

MMR protein expression in tumor tissue may be presented and analyzed using immunohistochemistry staining. The four most commonly defective MMR proteins (MLH-1, MSH-2, MSH-6, and PMS-2) can be analyzed [28,42]. There are four most common abnormal IHC patterns: the loss of MLH-1 and PMS-2, loss of MSH-2 and MSH-6, isolated loss of MSH-6, and isolated loss of PMS-2 [28,43]. The isolated loss of MLH-1 or MSH-2 expression is rare. To make diagnostics more cost-effective, a panel of only two proteins (MSH-6 and PMS-2) may be used [23,28,44]. However, some publications discourage this approach [43,45]. While performing IHC, it is possible to encounter difficulties during results interpretation. Especially in cases of obtaining an unusual staining pattern, interpretation should be performed by an experienced pathologist [46]. Weak or absent MMR protein expression is usually suspected to be secondary to technical problems. Thus, it may require comparison with an internal control and should be interpreted carefully. Moreover, the loss of MLH-1 protein expression may be a result of *MLH-1* gene promoter methylation without *MLH-1* mutation [28].

For MSI detection, a PCR-based five-microsatellite marker panel is usually used. This panel consists of markers such as BAT-25, BAT-26, MONO-27, NR-21, and NR-24 [47,48,49]. With the panel of five markers, samples can be characterized as MSI-High if mutations are found in at least two microsatellite markers or MSI-Low if mutations occur in one marker. The results of MSI analysis in tumors should be compared with the results of the examination of normal tissue (peripheral blood) from the same patients. The term microsatellite-stable (MSS) is used for samples with no mutated marker identified [21,48,49,50]. If the tumor is classified as MSS, then it is recommended to expand diagnostics for its proper characterization [50]. For larger MSI-NGS or MSI-PCR panels, different thresholds for MSI characterization are proposed. Tumors are usually considered MSI-H/MSI-positive if they have more than 20% [22,47] or approximately 30–40% markers unstable [47,51,52].

Although both MMR-IHC staining and MSI-PCR analysis are often considered equivalent, it is essential to remember that the optimality of their usage may vary, e.g., in different types of cancer [27]. It has been suggested that the usage of the molecular method alone is insufficient in EC patients, but it may be a valuable complement to IHC testing [42]. Considering the implementation of the technique, its accessibility and feasibility should be considered. Due to lower costs and a more accessible interpretation of IHC staining, it seems easier to apply in clinical practice than methods based on PCR or sequencing [44,53,54]. However, some authors suggest that two different approaches could be used for one patient to maximize sensitivity and minimize the risk of missing MMR dysfunction [28,42,46].

### 2.3. p53abn

p53 is a protein located in cell nuclei and cytoplasm. It is commonly called the “guardian of the genome” due to its crucial role in regulating gene expression, the cell cycle, and apoptosis [55,56]. Moreover, it is involved in stimulating and controlling damaged DNA repair systems [57,58]. A suppressor gene encodes p53–*TP53*. It is one of the most often altered genes in various cancers [55,59,60,61], including EC [15,62,63]. The clinical significance of *TP53* alterations and their impact on tumorigenesis is a very complex issue due to the diversity of those mutations [60,64]. p53abn is a subtype associated with poor prognosis and highest mortality among EC patients [16,20,62,65]. It is usually detected using IHC staining for abnormal p53 protein expression, but the determination of *TP53* pathogenic variants with NGS is also possible. Both strong and absent p53 protein expression in the IHC examination should be interpreted as p53abn [17,66].

#### p53abn Differentiation

EC is classified as a p53abn subtype if one of the abnormal p53 expression patterns, such as overexpression, null, or cytoplasmic, is observed in tissue staining. Among those patterns, overexpression occurs most frequently [66]. Tissue with a p53 wild-type pattern (i.e., normal p53 expression) shows the presence of negative cells and those with weak signals [67]. The mutant overexpression pattern is characterized as a strong nuclear expression of p53 in the majority (80 to 100%) of tumor cells and is mainly associated with a missense mutation in the *TP53* gene [66,67,68]. In the case of a null p53 expression pattern, the absence of staining for p53 is observed. The null pattern usually occurs in the presence of TP53 frameshift or nonsense mutations and large deletions in the *TP53* gene [66,68,69]. The cytoplasmic pattern is observed as diffused staining, localized mainly in the cytoplasm of cells due to the high expression of p53 in the cytoplasm. *TP53* tetramerization or C-terminal domain mutations lead to the presence of this pattern [66,67,70]. It is also possible to observe subclonal expression, i.e., the presence of two or more IHC patterns in the same tumor tissue sample (including a combination of wild-type and abnormal patterns or two or three abnormal patterns) [67,70]. EC is suggested to be classified as p53abn with the subclonal pattern if two or more staining patterns are present in at least 5% or 10% of tumor cells [70,71]. Interpreting subclonal patterns seems to be one of the most challenging issues during the performance of IHC staining [71].

Apart from IHC staining, NGS enables the determination of *TP53* mutation status [72]. Concordance between those methods has already been high [66,71,72], reaching up to approximately 95% [70,73]. Discrepancies in *TP53* status results obtained with different techniques may be an effect of difficulties in IHC interpretation, such as no assessment of positive internal control, the presence of artefacts, or tumor heterogeneity [73,74]. Moreover, it was suggested that the use of NGS may be beneficial in the case of indeterminate staining results [70,72].

### 2.4. POLEmut

Polymerase epsilon (Pol ε), among polymerases alpha and delta, is one of the crucial enzymes involved in DNA replication. It consists of four subunits. The largest subunit, p261, exhibits both catalytic and exonucleatic activity. p261 is encoded by the *POLE* gene [75,76]. During replication, Pol ε is involved in proofreading and, due to exonucleolytic activity, the replacement of mismatched bases [75,77]. Due to *POLE* gene mutations, the defective activity of Pol ε leads to increased molecular alterations.

In contrast to tumors with MMR deficiency, often called microsatellite-unstable and hypermutated (>10 mutations/Mb), tumors with Pol ε deficiency are mostly microsatellite-stable. Still, they harbor more mutations (>100 mutations/Mb) and are therefore characterized as ultra-mutated [77,78]. Although MMR may partially correct Pol ε deficiency, the activity of those proteins may be insufficient for highly effective mismatched base correction [75,77,79]. Moreover, mutations in *POLE* and genes encoding MMR proteins may coincide [75,77,80], contributing even more to mutation accumulation [80,81]. The *POLE*mut subtype is known to be associated with a favorable prognosis [16,82]. It was indicated that *POLE* mutations lead to the upregulation of the inflammatory response, including promoting cytotoxic lymphocyte recruitment. In comparison to wild-type *POLE* (*POLE*wt), samples with *POLE* mutations had higher amounts of cytotoxic T lymphocytes (CD3+/CD8+) [83]. Moreover, Bellone et al. observed that *POLE*mut EC is highly infiltrated with T helper lymphocytes (CD3+/CD4+) with PD-1 (programmed death 1) overexpression. Those results suggest that favorable outcomes of *POLE*mut patients may be associated with high tumor immunogenicity [84]. It is under consideration that, due to the impact on different signaling paths, *POLE* mutations may improve patients’ prognosis and be targets for possible new therapeutic approaches [82,83].

DNA sequencing enables the detection of pathogenic variants of the *POLE* gene and, therefore, the classification of EC as the *POLE*mut subtype. Tumors with nonpathogenic variants are not classified as *POLE*mut. However, during the analysis of 359 cases, McAlpine et al. identified most (82%) *POLE* mutations as being pathogenic [85]. The most frequent pathogenic mutations of *POLE* are located in 11 loci across 9, 11, 13, and 14 exons [86]. Among them, substitutions P286R, V411L, S297F, A456P, and S459F were indicated by León-Castillo et al. as being the most commonly occurring, while mutations in the six remaining domains (i.e., M295R, F367S, D368Y, L424I, P436R, and M444K) were rarer [86,87] (Table 1).

The *POLE*mut subtype is associated with excess genome substitutions, especially transversions (G:C>T:A). Church et al. showed that the highest rate of substitutions occurred in samples with the P286R variant, while substitutions were fewer in the presence of the S297F, A456P, and V411L variants [88]. These results are consistent with a Shinbrot et al. study in which it was pointed out that while P286R and S459F substitutions lead to the inactivation of Pol ε proofreading, the presence of the V411L variant does not cause a complete loss of this function but a decrease in its activity [89]. However, different studies have proven that pathogenic *POLE* variants may affect the proofreading capability of Pol ε, as well as its exonuclease activity and polymerase properties [90,91]. Tian et al. showed that P286R and V411L variants are associated with higher a Pol ε activity than *POLE*wt [90].

#### POLE Mutations Testing

Currently, *POLE* mutations are mostly determined by methods based on DNA sequencing, such as Sanger and next-generation sequencing [23,24,86,92]. Sanger sequencing preferentially amplifies the normal sequence of the *POLE* gene. Therefore, it may give false-negative results. NGS is expensive, takes a relatively long time to obtain results, and requires specialized personnel for proper analysis [24,86]. Therefore, new methods of *POLE* testing are under scientific investigation [93].

QPOLE is a method based on the performance of three quantitative PCR assays proposed by Van den Heerik et al. The first assay, QPOLE-frequent, was designed to determine five frequently occurring variants (P286R, V411L-T/C, S297F, A456P, and S459F). QPOLE-rare-1 and QPOLE-rare-2 enable the detection of, respectively, five rarer mutations (M295R, F367S, D368Y, L424I, and M444K) and P436R substitutions. QPOLE is 98.6% accurate and in concordance with NGS results. Moreover, QPOLE is cheaper and faster than NGS and seems easy to implement in clinical usage. The authors propose to perform all three assays, but it is possible to start with QPOLE-frequent and perform QPOLE-rare-1 and -2 only in cases of negative results for the five most common mutations [86].

The utility of droplet digital PCR (ddPCR) in *POLE* testing was also suggested. Even though ddPCR seems to be a promising tool for the detection of the five most frequent *POLE* pathogenic variants, the limitation of the conducted studies was the small number of tested samples [94,95]. Therefore, even though ddPCR usage seems to be a cost-effective and convenient sequencing alternative for detecting *POLE* mutations, it still requires expanded research.

Yu et al. proposed BaseScope, an in situ hybridization (ISH) assay for analyzing *POLE* variants in mRNA. The authors focused on the detection of the two most frequently occurring variants. The ISH assay detected P286R and V411L with high sensitivity and specificity (95% and 100%, respectively). It was possible to detect those variants simultaneously using mixed probes. However, further research is needed to determine whether other *POLE* mutations can be detected with ISH [96].

### 2.5. Multiple Classifiers

It is important to remember that a small percentage of EC patients (approximately 3–6%) may harbor more than one genetic condition. These cases are called “multiple classifiers” [16,97]. De Vitis et al. suggested that the percentage of “multiple classifiers” may be even higher (about 11% of EC cases) if, instead of p53 immunostaining, *TP53* mutation analysis is performed [98]. 

**Figure 2:**
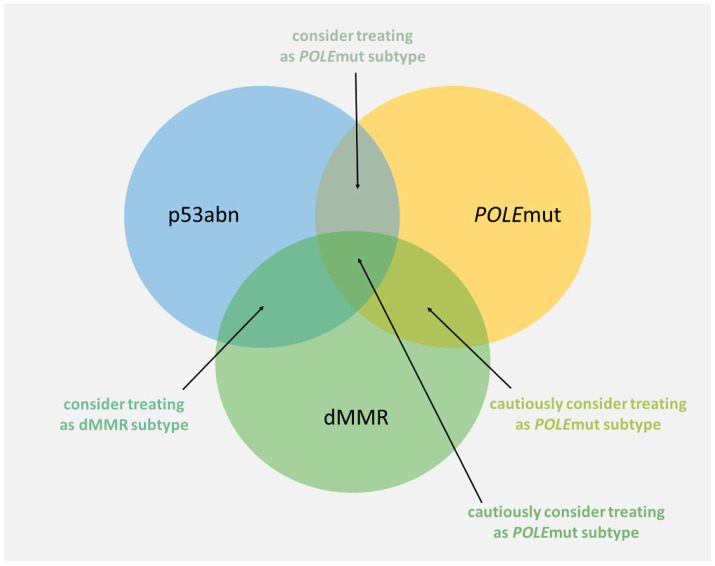
Proposed approach to therapy decision-making in multiple classifiers. Based on [97,98,99].

The most problematic aspect of therapy decision-making seems to be the abnormal expression of p53 and *POLE* mutations; thus, the p53abn subtype is known as a subtype with a poor prognosis, while *POLE*mut has a favorable prognosis. However, it has been suggested that patients with *POLE*mut-p53abn endometrial cancer have outcomes similar to the *POLE*mut subtype and, as a result, they should be treated as patients with the *POLE*mut subtype [16,98,99,100]. Similarly, it is suggested that patients with MMR deficiency and abnormal p53 expression should be classified as the dMMR subtype [16,100]. Information on the simultaneous occurrence of *POLE*mut and dMMR (including triple-classifiers, i.e., dMMR-*POLE*mut-p53abn subtype) is scarce and should be considered cautiously [16,97]. It was tentatively propounded to classify dMMR-*POLE*mut patients as *POLE*mut if a pathogenic *POLE* mutation is detected using NGS or if the mutation corresponds to one of the eleven most common pathogenic *POLE* variants [97] (Figure 2).

## 3. EC Treatment

The traditional approach for EC treatment is a combination of surgery and adjuvant therapy in the majority of non-metastatic cases [18]. Decisions about initiating and selecting adjuvant management should be based on clinical and pathological data, including tumor stage and grade [101]. However, the recognition of molecular subtypes should be incorporated into therapy decisions in all EC patients as well, especially in those with high-grade tumors. The outlook of EC management is evolving, including the complementation of conventional adjuvant treatment with targeted therapy, depending on the tumor molecular subtype. Moreover, new treatment methods have been introduced for recurrent or metastatic EC patients. [17,18,102]. Clinical trials aimed at optimizing personalized therapy are ongoing [103,104,105,106].

### 3.1. Standard Method of Endometrial Cancer Treatment before the Introduction of EC Molecular Classification

Endometrial cancer is usually treated with surgery, which involves total extrafascial hysterectomy with bilateral salpingoophorectomy. Open or minimally invasive approaches can do this. Minimally invasive procedures are recommended wherever possible [107,108]. Lymphadenectomy has no prognostic significance in endometrial cancer [109] but should be considered in patients with a significant (high–intermediate and high) risk of metastases in radiologically unchanged lymph nodes. Sentinel lymph node biopsy is increasingly used as an alternative to lymphadenectomy. However, the necessary condition is the ability to perform ultrastaging of the resected lymph nodes [110]. Infracolonic omentectomy should only be performed in patients with serous or undifferentiated EC [111].

In the new era of EC molecular classification, adjuvant treatment depends on the group of individuals at risk of recurrence. Only observation is recommended for patients after surgery with no residual disease or FIGO (Federation of Gynecology and Obstetrics) stage I or II of the low-risk group. Brachytherapy is recommended for patients of intermediate and high–intermediate risk groups, and in the high-risk group, brachytherapy and external beam pelvic radiotherapy (EBRT) are indicated [112]. Radiochemotherapy is an adjuvant treatment of choice in patients with FIGO stage III without residual disease [113]. Inoperable or locally advanced EC (FIGO IIIA/B/C/IVA) should be treated with EBRT and brachytherapy. Systemic treatment is indicated for incompletely operated EC or FIGO stage IVB [114]. Patients with low-grade estrogen- and progesterone-receptor-positive tumors and metastatic disease are treated with hormonal therapy, and standard first-line chemotherapy (carboplatin and paclitaxel) is administered for all other cases [115].

The new molecular classification of EC influenced not only surgical procedures and impacted the choice of adjuvant treatment but also identified new targeted therapies, which helped to individualize treatment.

### 3.2. Possible Treatment Options in Different Molecular Subtypes of EC

#### 3.2.1. NSMP

As mentioned before, NSMP subtype is a heterogeneous group of EC cases, and due to the limited ability of its stratification, the optimal therapeutic management may be especially problematic to determine [17,31]. Adjuvant radiotherapy and chemoradiotherapy are being used. However, due to the higher toxicity of chemoradiotherapy compared to radiotherapy alone [116], and the noticeable but uncertain benefits of this combined treatment in NSMP patients [33], alternative and less toxic options are being sought [103].

One of the widely used therapeutic paths for EC patients is hormonal therapy. It is considered adjuvant therapy in patients with low-grade EC without progressive disease [102]. However, Jamieson et al. pointed out that, despite its wide usage, studies on determining EC patients’ responses seem sparse [17]. The NSMP-ORANGE Trial (NCT05255653-3), part of the RAINBO program, may provide information on the potential clinical significance of this treatment method. This trial is focused on the comparison of the response to radiation combined with progestin to chemoradiotherapy and includes patients with the NSMP subtype and a high expression of estrogen receptors [103]. Moreover, a potential benefit for NSMP patients may be a combination of hormonal treatment and targeted agents. In a Mirza et al. study, the utility of a letrozole and palbociclib (inhibitor of cyclin-dependent kinases 4 and 6) combination in estrogen-receptor-positive patients with advanced or recurrent EC was suggested. It was indicated that this drug combination was associated with a statistically significant improvement in progression-free survival (PFS) [117].

#### 3.2.2. dMMR

It has already been indicated that cancers with MMR deficiency, including EC, may be susceptible to programmed death 1/programmed death-ligand 1 (PD-L1) inhibitors [106,118,119,120,121]. PD-L1 expression on tumor and/or immune cells is considered a predictive factor of immunotherapy efficacy in, e.g., non-small-cell lung cancer patients [121,122]. It was shown that PD-L1 expression is also frequently high in EC patients [123,124]. MSI-High and *POLE*mut tumors seem to be associated with PD-L1 overexpression on immune cells [124,125,126]. Moreover, studies have shown that dMMR subtypes, particularly high-grade tumors, are associated with high PD-L1 expression on tumor cells. The MMR-deficient tumor subtype is characterized by a high number of somatic mutations and high tumor mutation burden (TMB). Therefore, neoantigen accumulation on tumor cells is observed. These tumors become highly immunogenic. Thus, immune checkpoint inhibitors (ICIs), such as pembrolizumab and dostarlimab, are approved for the treatment of advanced or recurrent EC with dMMR or MSI-H [123,124].

However, adjuvant treatment options for EC patients with dMMR subtypes are still being investigated. Many clinical trials were performed and are still ongoing to better stratify the benefits of adjuvant immunotherapy in the dMMR EC cohort [18,101]. It has already been suggested that patients with MMR deficiencies may particularly benefit from the use of radiotherapy and immunotherapy combinations as adjuvant treatment [127]. The dMMR-GREEN Trial (NCT05255653-2) is intended to compare the efficacy of radiotherapy alone with radiotherapy in combination with durvalumab (PD-L1 inhibitor) [103]. PHAEDRA (NCT03015129) was another study on immunotherapy’s utility as an adjuvant therapy option. In this trial, EC patients were treated with durvalumab. Encouraging results of this therapeutic approach were observed in dMMR but not in the pMMR (proficient mismatch repair) cohort [128].

The real breakthrough came in treating advanced EC patients with MMR deficiency. The GARNET trial (NCT02715284) is focused on advanced or recurrent EC patients with dMMR or MSI-H who had disease progression after platinum-based chemotherapy. The control arm consisted of pMMR or MSS EC patients. The patients received dostarlimab in monotherapy. In patients with dMMR or MSI-H, the complete and partial response rate was 12.7% and 29.6%, respectively. In total, 57.7% of patients had disease control. The median duration of response was not reached, and the median PFS was six months. Additionally, 46.4% and 40.1% of patients remained without progression during 12 and 36 months of observation. These results were sufficient for dostarlimab approval for previously treated patients with the dMMR subtype of advanced or recurrent EC [106].

The efficacy and safety of chemotherapy based on carboplatin, paclitaxel, and immunotherapy are also being assessed during clinical trials. In the RUBY trial (NCT03981796), AtTEnd trial (NCT03603184), and NRG-GY018 trial (NCT03914612), the efficacy of first-line dostarlimab, atezolizumab, and pembrolizumab in combination with chemotherapy was analyzed in advanced or recurrent EC patients [104,129,130]. In the RUBY trial, the outcome of patients treated with chemotherapy based on carboplatin and paclitaxel and immunotherapy with dostarlimab was compared to chemotherapy with a placebo. The results of the RUBY trial have proven that dostarlimab, carboplatin, and paclitaxel treatment improved EC patients’ outcomes, especially in those with the dMMR subtype. Both a lower risk of death and disease progression were observed. In MMR-deficient patients, the median PFS was not reached in patients treated with chemoimmunotherapy, and it was 7.7 months in patients receiving only chemotherapy. Two-year PFS was observed in 61.4% and 15.7% of patients, respectively. The reduction in progression risk was 72% (HR = 0.28). However, in patients with pMMR and MSS tumors, the medians of PFS were 9.9 and 7.9 months and the reduction in progression risk was only 34% (HR = 0.76). The median overall survival (OS) was not reached in all groups of patients. However, the reduction in death risk was 70% (HR = 0.30) in the group of dMMR/MSI-H patients and 27% (HR = 0.73) in the pMMR/MSS patient group. In the group of MMR-deficient patients, 88.3% of patients treated with chemoimmunotherapy and 55.1% of patients receiving chemotherapy were still alive after two years of observation. In this group of patients, complete response (CR) and partial response (PR) were observed in 22.9% and 54.2% of patients treated with dostarlimab and chemotherapy and in 13.3% and 50% of patients receiving chemotherapy alone [104].

In the Phase III AtTEnd and NRG-GY018 trials, the immunotherapeutic agent was observed to be added to chemotherapy based on carboplatin and paclitaxel, which was associated with a longer progression-free survival. Analogously to the RUBY trial, in the AtTEnd and NRG-GY018 trials, the most significant benefits were obtained in MMR-deficient patients, although the outcome was also improved in pMMR patients [129,130].

Even though dMMR is considered an EC subtype with an intermediate prognosis, ongoing research and numerous potential therapeutic targets provide hope for even more efficient treatment methods. For example, a combination of lenvatinib (multikinase inhibitor) with pembrolizumab seems to be advantageous in recurrent EC cases [105].

With the complete publication in early 2024, the DUO-E trial provided additional data in the advanced/metastatic setting. Patients were randomized to first-line treatment with 6 cycles of CP only or combined with durvalumab with or without olaparib. The study showed the benefit of adding durvalumab in the dMMR/MSI population, while the optimal olaparib indication in different subtypes remains unclear [131].

#### 3.2.3. p53abn

It has already been shown that adjuvant therapy with chemotherapy and radiation is associated with significant benefits in EC patients with the p53abn subtype [33]. Although p53abn EC prognosis remains poor [16,33], different therapeutic approaches are being studied to improve patients’ outcomes. The addition of anti-HER2 (human epidermal growth factor receptor 2) antibodies, poly ADP ribose polymerase (PARP) inhibitors, immune checkpoint inhibitors, or anti-angiogenic agents to chemotherapy is being proposed [17,132].

A combination of chemotherapy and anti-HER2 antibodies seems to be one of the possibly beneficial therapeutic paths in EC patients with the p53abn subtype. Fader et al. indicated that trastuzumab, in addition to chemotherapy based on carboplatin and paclitaxel, is associated with increased progression-free survival and overall survival in HER2-positive patients with an aggressive EC subtype—uterine serous carcinoma [133,134]. Vermij et al. showed that HER2 positivity mostly coexisted with the p53abn subtype and suggested that patients classified as the p53abn subtype should be tested to determine HER2 status [135]. Clinical trials are still being performed to evaluate the efficacy of anti-HER2 antibodies in monotherapy and in combination with chemotherapy or anti-HER2 and cytostatic conjugates [132,136]. However, The National Comprehensive Cancer Network has already recommended trastuzumab (anti-HER2 antibody) as an addition to chemotherapy in patients with recurrent or advanced HER2-positivie serous EC [137].

Different possible therapeutic approaches include the addition of PARP inhibitors [103] because it is considered that cancers with homologous recombination deficiency (HRD) are more sensitive to PARP inhibitors [17]. HRD may occur in EC patients, especially in *TP53* mutated tumors. In a de Jonge et al. study, 46% of *TP53* mutated EC also had HRD. Therefore, PARP inhibitors are considered beneficial, particularly in the p53abn subtype of EC [138]. The efficacy and toxicity of olaparib (PARP inhibitor) and chemoradiotherapy combination compared to chemoradiotherapy alone will be assessed during the p53abn-RED trial (NCT05255653-1) [103].

During the preplanned subanalyses of the RUBY trial, it was found that both the PFS and OS benefits of the addition of dostarlimab to carboplatin and paclitaxel are not restricted to the dMMR/MSI population but are also clearly pronounced in the p53abn subgroup. These findings must be proven in a prospective trial, and a biological explanation of this phenomenon is highly expected [139].

Another possible option in treating patients with the p53abn subtype is using an anti-angiogenic agent, e.g., bevacizumab. It was suggested that bevacizumab, in addition to chemotherapy, does not improve EC patients’ outcomes in general [140]. However, Leslie et al. showed that this combination seems beneficial in the p53abn subtype compared to treatment with chemotherapy and temsirolimus. Moreover, the authors proposed *TP53* mutation as a potential biomarker of sensitivity to bevacizumab in EC patients [141]. Due to unfavorable outcomes of patients with the p53abn subtype, a better stratification of the aforementioned drugs’ efficacy or an indication of other therapeutic paths seems especially crucial.

#### 3.2.4. *POLE*mut

The consideration of adjuvant treatment omission in *POLE*mut patients at an early disease stage (stage I–II and low-risk) has already been proposed in ESGO/ESTRO/ESP guidelines published in 2020 [102]. Scientific research indicated that adjuvant therapy does not improve the outcome of patients with the *POLE*mut subtype [85,103,142]. The meta-analysis by McAlpine et al. suggested that a favorable *POLE*mut prognosis is independent of implemented adjuvant therapy, at least for stages I–II of the disease. However, stage III and IV results were not specific [85]. Research performed on tumor cells with *POLE* mutations in an in vitro model suggested that this mutation is not associated with increased sensitivity to radiotherapy or commonly used cytotoxic drugs. Moreover, in this study, Van Gool et al. proposed nucleoside analogues (e.g., cytarabine and fludarabine) as a potential alternative in *POLE*-mutated cancer treatment. However, scientific research must be much more extensive to confirm the possibility of this drug’s usage in this cohort of patients [142]. On the other hand, further studies, mainly performed on patients with advanced-stage EC, will be valuable to more accurately assess whether patients with the *POLE*mut subtype should be offered the omission of adjuvant treatment [85,143].

The *POLE*mut-BLUE Trial (NCT05255653-4) is designed to assess the safety of de-escalation treatment in patients with the *POLE*mut subtype. In this trial, no adjuvant treatment is implemented in patients with low-risk diseases, whereas in those with high-risk disease, no adjuvant treatment or radiotherapy is used [103]. The results of the PORTEC-3 Trial (NCT00411138) have already suggested that adjuvant treatment de-escalation should be considered in *POLE*mut patients. However, attention was also paid to the importance of further patient observations, which will be beneficial in stratifying the adequacy of this therapeutic approach [33]. The PORTEC-4a Trial (NCT03469674), designed to compare different adjuvant therapeutic approaches (therapy based on molecular classification versus radiotherapy), will also include *POLE*mut patients. Among others, the safety of adjuvant therapy in patients with favorable prognoses will be analyzed [93].

## 4. Conclusions

The molecular classification of EC has already been proven as a valuable tool guiding optimal therapeutic management and supporting patients’ prognosis stratification. The subtyping of all EC cases has been recommended in international guidelines. Following those recommendations, even though molecular classification requires immunohistochemistry and sequencing implementation, it should not be missed or underestimated during therapy decision-making. Moreover, various analyses and clinical trials are being performed, suggesting new, promising paths in endometrial cancer treatment dependent on EC subtype.

## Figures and Tables

**Table 1:** Characterization of *POLE* gene pathological variants. Based on [86,87].

**Most Frequent Variants of *POLE* Gene in Endometrial Cancer**		
**Protein Change**	**Nucleotide Substitution**	**Exon**
P286R	c.857C>G	9
V411L	c.1231G>C c.1231G>T	13
S297F	c.890C>T	9
A456P	c.1366G>C	14
S459F	c.1376C>T	14
**Least Frequent Variants of the *POLE* Gene in Endometrial Cancer**		
**Protein Change**	**Nucleotide Substitution**	**Exon**
M295R	c.884T>G	9
F367S	c.1100T>C	11
D368Y	c.1102G>T	11
L424I	c.1270C>A	13
P436R	c.1307C>G	13
M444K	c.1331T>A	13

## Data Availability

Not applicable.
